# Vascular smooth muscle ion channels in essential hypertension

**DOI:** 10.3389/fphys.2022.1016175

**Published:** 2022-09-23

**Authors:** Nuria Daghbouche-Rubio, José Ramón López-López, María Teresa Pérez-García, Pilar Cidad

**Affiliations:** Departamento de Bioquímica y Biología Molecular y Fisiología and Instituto de Biología y Genética Molecular (IBGM), Universidad de Valladolid and Consejo Superior de Investigaciones Científicas (CSIC), Valladolid, Spain

**Keywords:** ion channels, hypertension, vascular smooth muscle cells, BPH mice, membrane potencial, vascular remodeling

## Abstract

Hypertension is a highly prevalent chronic disease and the major risk factor for cardiovascular diseases, the leading cause of death worldwide. Hypertension is characterized by an increased vascular tone determined by the contractile state of vascular smooth muscle cells that depends on intracellular calcium levels. The interplay of ion channels determine VSMCs membrane potential and thus intracellular calcium that controls the degree of contraction, vascular tone and blood pressure. Changes in ion channels expression and function have been linked to hypertension, but the mechanisms and molecular entities involved are not completely clear. Furthermore, the literature shows discrepancies regarding the contribution of different ion channels to hypertension probably due to differences both in the vascular preparation and in the model of hypertension employed. Animal models are essential to study this multifactorial disease but it is also critical to know their characteristics to interpret properly the results obtained. In this review we summarize previous studies, using the hypertensive mouse (BPH) and its normotensive control (BPN), focused on the identified changes in the expression and function of different families of ion channels. We will focus on L-type voltage-dependent Ca^2+^ channels (Cav1.2), canonical transient receptor potential channels and four different classes of K^+^ channels: voltage-activated (Kv), large conductance Ca^2+^-activated (BK), inward rectifiers (Kir) and ATP-sensitive (K_ATP_) K^+^ channels. We will describe the role of these channels in hypertension and we will discuss the importance of integrating individual changes in a global context to understand the complex interplay of ion channels in hypertension.

## Introduction

Hypertension is one of the most frequent chronic diseases worldwide, affecting more than 30% of the total adult population with an incidence increasing globally. It is the major preventable risk factor for cardiovascular diseases, which are the leading cause of premature death and disability in the western countries (Mills et al., 2020). Essential hypertension accounts for 95% of human hypertension and is a heterogeneous condition of unknown etiology resulting from the complex interaction of multiple genetic and environmental factors that involves multiple organs and systems (Messerli et al., 2007). In any case, it is characterized by an increased vascular tone that leads to an increase in the total arterial peripheral resistance (Joseph et al., 2013).

## Ion channels and vascular tone

**FIGURE 1:**
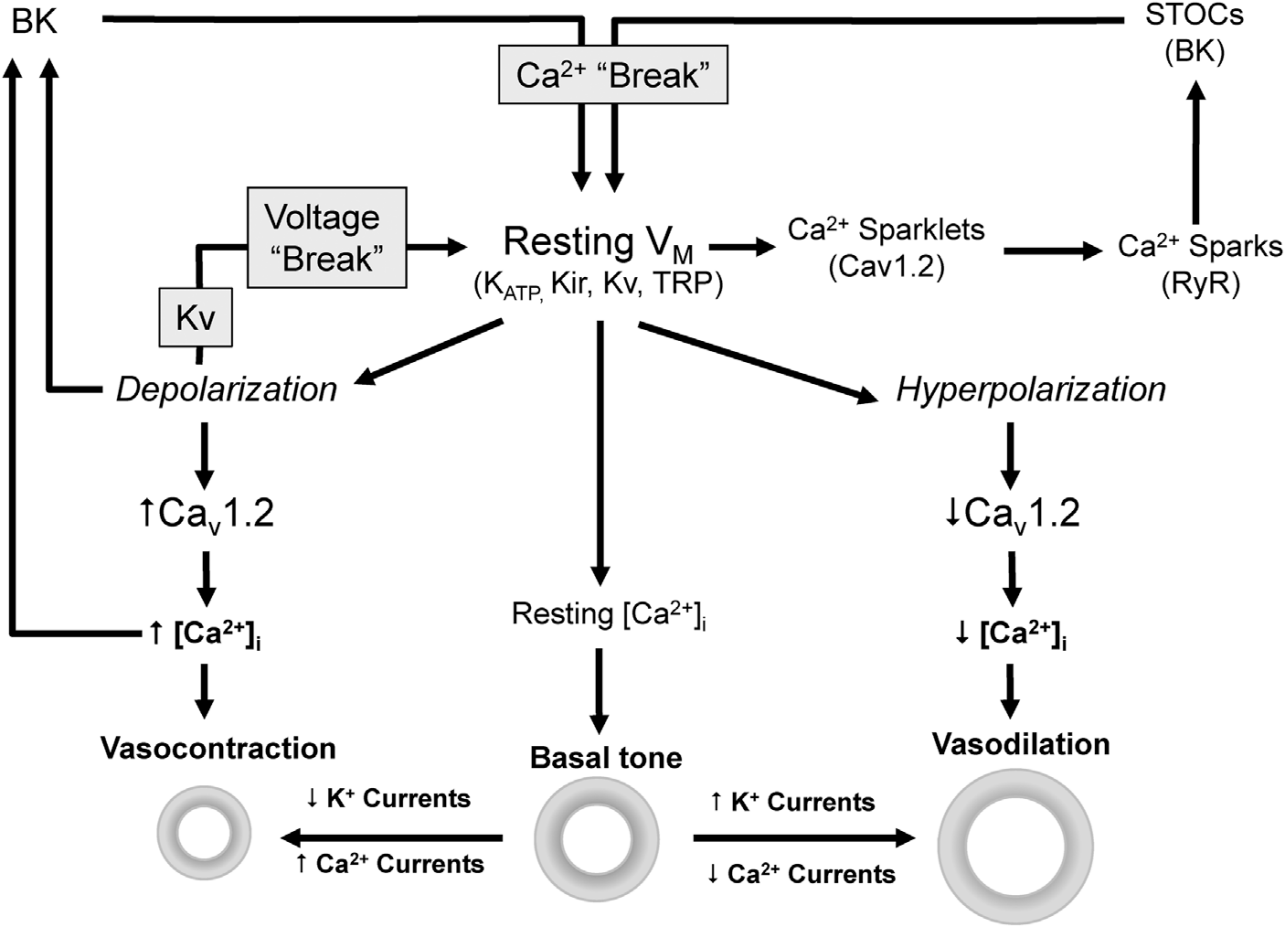
Ion channels modulate V_M_ [Ca^2+^]_i_ and vascular tone. Vascular tone depends on [Ca^2+^]_i_ which is mainly determined by the V_M_ dependent activity of Cav1.2. Depolarization activates Cav1.2, increases Ca^2+^ influx and [Ca^2+^]_i_ leading to vasoconstriction. On the contrary hyperpolarization leads to closure of Cav1.2 channels and ultimately to vasodilation.

Vascular tone depends on the integrated contractile response of VSMCs to many vasodilator and vasoconstrictor stimuli. The level of contraction determines the diameter and the resistance of the blood vessel. However, independently of the stimuli, contraction is ultimately dependent on an increase in the intracellular calcium concentration [Ca^2+^]_i_ and the activation of Ca^2+^/Calmodulin-dependent myosin light chain kinase (Jackson, 2000). The major pathways for this increase are the influx through voltage-dependent Ca^2+^ channels (VDCCs) and non-selective cation channels at the plasma membrane, or the Ca^2+^ release from intracellular stores. However, global [Ca^2+^]_i_ is mainly determined by the open probability VDCCs, which is controlled by membrane potential (V_M_) (Figure 1). Consequently, any factor that modulate V_M_ have a direct impact on global [Ca^2+^]_i_, contraction, vascular resistance and blood pressure (Nelson et al., 1990; Nelson & Quayle, 1995; Cox & Rusch, 2002; Ledoux et al., 2006). On the other hand, local Ca^2+^ transients tightly regulate V_M_ modulating the activity of Ca^2+^-dependent K^+^ channels (Figure 1). Cav1.2 are the main VDCCs of VSMCs. Their spontaneous activity at resting V_M_ produce local Ca^2+^ transients named “Ca^2+^ sparklets” (Santana et al., 2008) that activate ryanodine receptors (RyR) in the sarcoplasmic reticulum (SR) leading to the release of Ca^2+^ and the production of a larger local transients named “Ca^2+^ sparks”. In VSMCs, Ca^2+^ sparks have a local spatial spread with minimal direct impact in global [Ca^2+^]_i_ but big effect on V_M_ by activating BK channels, generating spontaneous transient outward currents (STOCs) and leading to a hyperpolarization that keeps Cav1.2 open probability low (Jaggar et al., 1998; Wellman & Nelson, 2003).

## Vascular remodeling in hypertension

Chronic hypertension leads to structural and molecular changes in small arteries and arterioles in response to the elevated intraluminal pressure (Lehoux et al., 2006; Anwar et al., 2012). Among these changes, VSMCs undergo an “electrical remodeling” thereby changes in the expression of ion channels generate a disease-specific expression profile that contribute to set an increased vascular tone. In this remodeling, changes contributing to increase [Ca^2+^]_i_ coexist with adaptive responses aimed to counteract the pro-hypertensive changes (Joseph et al., 2013). The relationships between hypertension and VSMCs ion channel are complex, and their classification as cause or consequence of the altered vascular tone is not always clear.

VSMC depolarization at rest is a common feature described in several experimental models of hypertension (Nelson & Quayle, 1995; Cox & Rusch, 2002; Joseph et al., 2013). Downregulation of K^+^ channels together with increased Cav1.2 function and increased compensatory overexpression of BK channels has been proposed as a possible mechanism (Cox & Rusch, 2002). However, the underlying molecular mechanisms are poorly defined. The large diversity of ion channels present in VSMCs, the existence of vascular-bed specific patterns of expression and the use of different species and experimental models have made difficult this characterization (Coetzee et al., 1999; Harder, 1983; Sobey, 2001; Tajada et al., 2012; Tykocki et al., 2017).

## Genetic model of essential hypertension: Schlager BPH mice

As a complex, multifactorial and systemic disease that involves multiple organs as systems, an important challenge is the use of an adequate model that emulates all of the components that contribute to the phenotype of essential hypertension. There are genetic and non-genetic models (Jama et al., 2021) but here we will focus on a mouse model of genetic hypertension: the Schlager BPH mice.

These mice were obtained by the phenotypic selection of the natural variants with higher pressures after crossbreeding of eight different strains. This approach established three inbred lines sharing genetic background with low (BPL), high (PBH) and normal (BPN) blood pressure (Schlager, 1974). This model shares many features with human hypertension, some of them common to another genetic model, the spontaneously hypertensive rat (SHR, Friese et al., 2005). BPH mice show a mild elevated BP from as young as 6 weeks and with the maximal divergence at 21 weeks. They also show increased heart rate, lower body weight and a reduced lifespan when compared to BPN mice (Schlager and Sides, 1997; Jackson et al., 2019). Numerous evidences point to a predominantly neurogenic mechanism of hypertension, with increased activity of the sympathetic nervous system, which in the kidney will lead to enhanced renin synthesis (Jackson et al., 2013, 2019; Gueguen et al., 2019). BPH mice also present global metabolic abnormalities, enhanced oxidative stress and alterations in elements of the mitochondrial electron transport chain, which could be relevant to metabolism and ROS production (Friese et al., 2005).

The characteristics of this model, and particularly the existence of a control strain with a similar genetic background (the BPN strain), makes BPH an attractive model to study essential hypertension.

## Vascular remodeling in schlager BPH mice

Hypertension is usually associated in resistance vessels with an inward eutrophic remodeling where the same number of cells reorganize themselves around a smaller diameter (Mulvany, 2002). However, in BPH mice, the mesenteric vessels show outward hypertrophic remodeling, with larger lumen size and wall thickness because of an increased VSMCs size (Moreno-Domínguez et al., 2009). This could be due to an exposure to increase flow because of the reduction of parallel-connected vessels (rarefaction), as high flow leads to hypertrophy (Mulvany, 2002). In fact, outward hypertrophy remodeling has been described in rat mesenteric arteries exposed to high flow *in vitro* (Buus et al., 2001).

**FIGURE 2:**
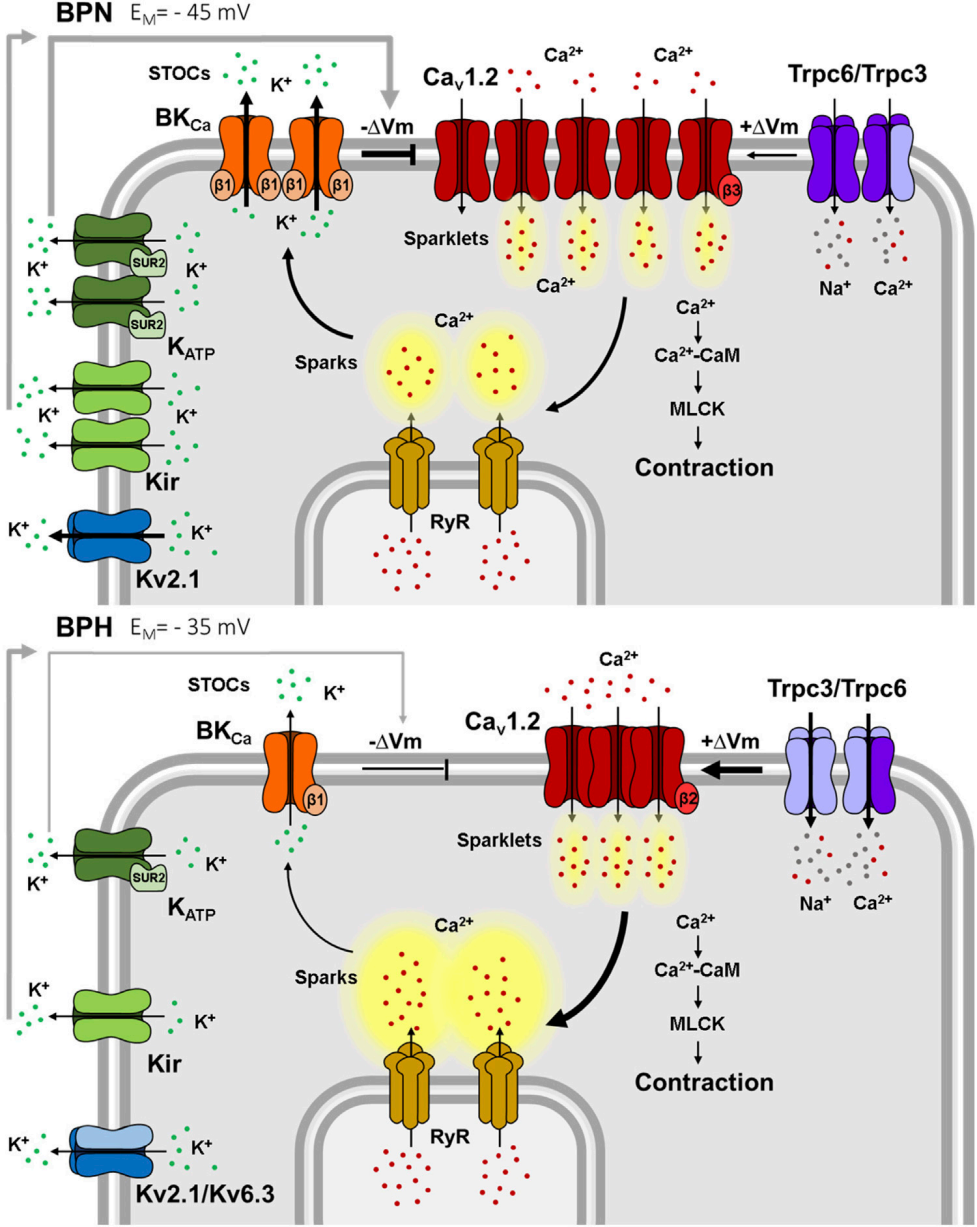
Ion channels differences between mesenteric BPN and BPH VSMCs. Smaller K^+^ currents in BPH cells lead to a depolarized resting V_M_. Kv2.1 currents are smaller because of the “*de novo*” expression of the Kv6.3 subunit. Kir, K_ATP_ and BK channels functional expression is smaller, and BK have a decreased sensitivity to Ca^2+^ due to the decreased expression of the BKβ1 subunit. BPH VSMCs also have a higher expression of TRPC3 and a different composition of the TRPC3/TRPC6 heterotetramers. Larger TRPC currents contribute to the depolarized resting V_M_. Surprisingly, Cav1.2 expression and total Ca^2+^ currents are smaller in BPH, but the different expression of β subunits generate clusters of channels that produce higher Ca^2+^ sparklets and induce larger RyR Ca^2+^ sparks. However, these larger sparks do not induce larger STOCs, due to the reduced Ca^2+^ sensitivity of BK channels, jeopardizing the “Ca^2+^ break”. Ca^2+^ (red dots), K^+^ (green dots) and Na^2+^ (grey dots) ions.

Depolarization of VSMCs is a hallmark of hypertension reported in different models and vascular beds and it has been associated with an enhanced myogenic tone in arteries from hypertensive animals (Harder et al., 1983; Harder et al., 1985; Cox & Rusch, 2002). Mesenteric VSMCs from BPH show depolarized resting V_M_ values, and mesenteric arteries exhibit higher myogenic tone than BPN (Moreno-Domínguez et al., 2009; Tajada et al., 2012). The electrical remodeling responsible of resetting resting V_M_ is complex and it has been comprehensively analyzed in mesenteric arteries of BPH mice. This remodeling is the focus of this review (Figure 2).

## K^+^ channels remodeling

K^+^ channels are key players in setting resting V_M_. Their activation hyperpolarize VSMCs, decreasing the open probability of VDCCs and Ca^2+^ entry, promoting vasodilation. On the contrary, the closure of K^+^ channels depolarizes VSMCs, opens VDCCs, increases Ca^2+^ influx and promotes vasoconstriction. Therefore, a plausible hypothesis to explain VSMCs depolarization in hypertension postulates the existence of a decreased expression of K^+^ channels. In fact, since the pioneering studies reporting an abnormally low permeability of the plasma membrane to K^+^ ions in VSMCs of cerebral arteries from hypertensive animals (Harder et al., 1983), a loss of resting K^+^ efflux resulting in depolarization is a common finding in VSMCs from different vascular beds (as mesenteric or cremaster arteries) under high BP (Sonkusare et al., 2006). Several types of K^+^ channels have been described in VSMCs, and their contribution to the hypertensive VSMCs has been characterized in different preparations.

**Voltage-dependent K^+^ (Kv) channels** are activated by membrane depolarization in the range of resting V_M_ values (around -35 to -45 mV) providing a negative feedback to depolarization (Jackson, 2018). Thus through their contribution to regulation of V_M_ they have a major influence on VDCCs activation and vascular tone. Among Kv channels, members of the Kv1, Kv2 and Kv7 subfamilies are particularly important regulating V_M_ in VSMCs. Decreased functional expression of Kv1 (Tobin et al., 2009), Kv2 (Amberg & Santana, 2006) and Kv7 (Jepps et al., 2011) are among the most common changes described in hypertension (Jackson, 2018; Nieves-Cintrón et al., 2018).

In BPH mesenteric VSMCs, both mRNA expression studies and electrophysiological recordings indicate that members of the Kv1 and Kv2 subfamilies (mainly Kv1.1, Kv1.2, Kv1.5, Kv1.6 and Kv2.1) are principal contributors to Kv currents (Moreno-Domínguez et al., 2009) in agreement with data from other preparations (Fountain et al., 2004; Plane et al., 2005; Amberg & Santana, 2006). However, while no differences in mRNA expression levels for Kv1 and Kv2 channels were observed between BPN and BPH VSMCs, a significant decrease of the contribution of Kv2 currents to total Kv currents was described in BPH cells. This change could be explained by the *de novo* expression of Kv6.3 channels. Kv6.3 channels do not form functional channels but co-assemble with Kv2 subunits to produce heterotetrameric channels with different kinetics and pharmacological properties, including a decrease in the current amplitude (Salinas et al., 1997; Vega-Saenz De Miera, 2004).

Both Kv1 and Kv2 currents modulate resting V_M_ in VSMCs, but no differences in their contribution between BPN and BPH were observed, suggesting that changes in Kv channels do not explain the depolarization of BPH mesenteric VSMCs (Moreno-Domínguez et al., 2009).

**Inward rectifying K^+^ channels (Kir and K_ATP_)** allow greater inward than outward K^+^ currents, and are active at more negative voltage than Kv channels (Nelson & Quayle, 1995; Bichet et al., 2003). In addition to V_M_, the external K^+^ concentration modulates Kir activity, and the small increases of K^+^ occurring during muscle activation, promotes Kir activation and then, vasodilation to increase muscle blood flow. They have been found in VSMCs from different resistance vessels (cerebral, renal interlobular and mesenteric arteries as well as cremaster and renal afferent arterioles) and it has been described that their blockade leads to depolarization and increased vascular tone (Tykocki et al., 2017). On the other hand, K_ATP_ channels are inhibited by intracellular ATP, linking cellular metabolism to V_M_ (Tykocki et al., 2017). Under normal ATP concentration, their activity should be low but they are open due to phosphorylation through the basal activity of protein kinase A (Ko et al., 2008). K_ATP_ channels are functional hetero-octomers composed of four pore-forming subunits (Kir 6.1 or Kir 6.2) and four regulatory subunits (the sulfonylurea receptors, SURx) that confer sensitivity to ATP. Kir6.1 and SUR2 are the predominant subunits in VSMCs (Hibino et al., 2010). A number of evidences suggest a reduced expression and function of Kir and K_ATP_ channels in hypertension although some discrepancy can be found in the literature (Sobey, 2001; Tykocki et al., 2017).

In VSMCs from BPH mesenteric arteries, there is a decreased mRNA expression of the most abundant Kir (Kir2.1, Ki4.1) and K_ATP_ channels (Kir6.1 and Sur2). There is also a significant decrease of both Kir and K_ATP_ current amplitudes. K_ATP_ currents are larger than Kir in BPN cells and are more downregulated in BPH VSMCs (Tajada et al., 2012). Both, Kir and K_ATP_ channels contribute to set the resting V_M_, and their contribution was significantly smaller in BPH cells. However, when exploring the contribution of Kir and K_ATP_ remodeling to set vascular tone in BPH arteries, only the changes in K_ATP_ were clearly relevant. These data suggest that changes in K_ATP_ channels in resistance arteries could be the principal determinant of VSMCs depolarization in hypertension (Tajada et al., 2012).

**Large-conductance Ca^2+^-activated channels (BK)** are the most abundant K^+^ channels in VSMCs and have been described in all vascular beds studied from large vessels to arterioles. BK channels exhibit a large unitary conductance and since they are activated by increases in [Ca^2+^]_i_ and/or V_M_ (Nelson & Quayle, 1995; Joseph et al., 2013) they play a central role in the regulation of vascular tone acting as a negative feedback mechanism. BK channels are comprised of four pore-forming α-subunits that coassemble with none to four regulatory β-subunits. Four β-subunits isoforms have been described, being β1 the main isoform in VSMCs that confers enhanced Ca^2+^ sensitivity to BK channels (Brenner et al., 2000; Ledoux et al., 2006). Recently a new regulatory subunit that increases voltage sensitivity to BK channels has been described, the γ-subunits (Evanson et al., 2014; Gonzalez-Perez & Lingle, 2019). Due to their close proximity to the SR, local Ca^2+^ transients elicited by Ca^2+^ release from RyR stimulate BK channels opening and the K^+^ efflux that limits vasoconstriction (Jaggar et al., 1998; Wellman & Nelson, 2003).

Related to the expression and function of BK in hypertension contradictory changes have been reported. Enhanced BK currents have been found in arteries form hypertensive rats, explained as a protective mechanism to limit vasoconstriction (Sobey, 2001; Cox & Rusch, 2002). However, reduced BK currents, with lower Ca^2+^ sensitivity, have also been described in other works (Amberg et al., 2003; Amberg & Santana, 2003), and the β1-knockout mouse has a hypertensive phenotype (Brenner et al., 2000).

In VSMCs from BPH mesenteric arteries, mRNA expression of BKα and β1 subunits is significantly downregulated. Accordingly, BK currents are smaller and exhibit a decreased sensitivity to Ca^2+^, so that the frequency and amplitude of STOCs are decreased (Moreno-Domínguez et al., 2009). Therefore, BK remodeling impairs the negative feedback elicited by STOCs on V_M_ and contributes significantly to the hypertensive phenotype.

## Non-selective cation channels remodeling

Non-selective cation channels of the TRP family have also been identified as important players in the regulation of vascular tone, either modulating membrane potential or providing a Ca^2+^ entry pathway independent of the activation of VDCCs (Albert & Large, 2006; Earley & Brayden, 2015). Among TRP channels, several members of the canonical TRP (TRPC) family have been proposed as the molecular constituents of the receptor-operated channels that link the PLC-DAG signaling cascade to the activation of VDCCs. Agonist binding to GPCRs stimulates PLC leading to DAG production that directly activates TRPC3/6/7 channels leading to cell depolarization (Hofmann et al., 1999). In VSMCs only TRPC3 and TRPC6 have been found (Earley & Brayden, 2015).

Numerous observations associate altered expression of TRPC3 and TRPC6 channels with hypertension in animal models. Several studies reported an increased expression of TRPC3 (Liu et al., 2009; Chen et al., 2010; Noorani et al., 2011) or TRPC6 channels (Zulian et al., 2010; Linde et al., 2012) which correlate with enhanced agonists-induced Ca^2+^ influx and contraction. Unexpectedly, TRPC6 knockout mice showed a hypertensive phenotype, which was explained by the compensatory upregulation of TRPC3 channels (Dietrich et al., 2005).

VSMCs from BPN mesenteric arteries express TRPC3 and TRPC6 channels and BPH VSMCs showed a larger expression of TRPC3 channels. Pharmacological dissection shows that BPH cells have larger non-selective cationic currents with higher contribution of TRPC3. BPH have a higher expression of TRPC3 in the membrane as homo- or heterotetramers with TRPC6, while TRPC6 homomultimers predominate in BPN. The larger expression of TRPC3 in BPH determines differences in the TRPC3/C6 proportion and assembly that favors cell depolarization in hypertension (Álvarez-Miguel et al., 2017). The increased TRPC3 expression determines an increased cation permeability at rest, contributing to the membrane depolarization of BPH cells.

## Ca^2+^ channels remodeling

Cav1.2 are the principal voltage-dependent Ca^2+^ channels and the primary Ca^2+^ influx pathway in VSMCs. These channels open by depolarization and close by hyperpolarization playing a central role in regulation of vascular tone by V_M_. Cav1.2 currents activate at relatively positive potentials (at -30 to -40 mV), have high single channel conductance and show slow voltage-dependent inactivation (Tykocki et al., 2017). They are multimeric complexes comprised of the pore-forming α1 subunit and three auxiliary subunits (β, α2δ and γ) arranged in 1:1:1:1 stoichiometry. The α1 subunit confers most of the functional properties to Cav1.2 channels, including voltage sensing, Ca^2+^ permeability and inhibition by channel blockers. The auxiliary subunits enhance channel expression and modulate biophysical and physiological properties (Catterall, 2000).

Upregulation of Cav1.2 is a generally accepted feature of hypertension that has been described in different animal models and arteries following elevated BP and/or VSMCs depolarization (Lozinskaya & Cox, 1997; Simard et al., 1998; Pratt et al., 2002; Pesic et al., 2004; Sonkusare et al., 2006). In contrast, VSMCs obtained from BPH mesenteric arteries showed a markedly decrease in whole cell Cav1.2 currents, with a lower mRNA and protein expression of the pore forming α1 subunit when compared with BPN cells (Tajada et al., 2013).

The influx of Ca^2+^ through single or clustered Cav1.2 channels can be optically detected as “Ca^2+^ sparklets” (Santana et al., 2008). While their amplitude was similar in BPN and BPH cells, they exhibited a higher frequency and higher density in BPH VSMCs. In spite of having smaller whole currents, the differences of “Ca^2+^ sparklets” indicate a more efficient clustering of Cav1.2 channels in BPH (Tajada et al., 2013; Dixon et al., 2022) that can be explained in part by the different composition of the Cav1.2 auxiliary subunits. Expression and functional studies both in native cells and in heterologous expression systems indicate that changes in the clustering (and hence the local activity and Ca^2+^ signaling through Cav1.2 channels) are consequence of the different composition of Cav1.2 channel complexes. In BPN, the Cav1.2 complexes are mainly α1/α2δ1/β3, while in BPH they are α1/α2δ1/β2. Cav1.2β subunits have been involved in the trafficking and membrane expression of Cav1.2 in VSMCs (Murakami et al., 2003; Dolphin, 2009), and in the regulation of the size and the density of Cav1.2 clusters at the plasma membrane (Kobrinsky et al., 2009). The β2 subunit in Cav1.2 complexes favors the formation of larger channel clusters with increased activity, in spite of the reduction in the total Cav1.2 currents. In the BPH cells, this higher Ca^2+^ sparklets activity triggers an increased Ca^2+^ release from SR, but these larger sparks do not produce larger STOCs (Tajada et al., 2013) (Figure 2). As described above, in BPH VSMCs, Ca^2+^ sparks are uncoupled from BK channel activation due to lower expression of the β1 subunit of BK (Moreno-Domínguez et al., 2009). The complex remodeling of Ca^2+^ and BK channels contribute to generate a hypertensive phenotype by increasing the basal activity of Ca2+ channels and impairing the negative feedback mechanisms that rely on the Ca^2+^-dependent activation of BKs.

## Conclusions and future perspectives

Hypertension is a complex and heterogeneous disease of unknown etiology. Blood pressure control involves many organs and systems, and in most of the cases, dysregulation is the result of many changes that contribute in a little percentage to the final output (Padmanabhan & Dominiczak, 2021)*.* Actually, from a mechanistic perspective, probably there are as many types of hypertension as there are hypertensive patients. Thus, the study of the mechanisms is very dependent on model, and it is of paramount importance to contextualize all changes associated with the hypertensive phenotype to weigh their functional relevance and their possible use as therapeutic targets.

In this regard, the BPH mice is a genetic phenotypic-driven model of mild hypertension that resembles a polygenic disease where no single genetic defect can explain the development of the disease (Lerman et al., 2005). In this model, the normotensive control shares a similar genotypic background, improving the strength of comparisons of the physiological changes related with the hypertensive phenotype. This is a clear advantage against other models, like the SHR rats, which do not seem to share the genetic background of the Wistar-Kyoto rats used as controls (Zhang-James et al., 2013).

The changes of VSMCs ion channels that associate with hypertension either contribute to increase vascular tone or behave as compensatory mechanisms to soften such increase. Although it is hard to state undoubtedly, an in depth characterization of the physiology of BPH and BPN can be used to dissect both types of changes, and the knowledge of their functional interplay is relevant to understand the role of a particular channel as a possible therapeutic target. For instance, in the case of K^+^ channels, while the reduced functional expression of BK channels in BPH mice contributes to maintain the hypertensive phenotype, the “*de novo*” expression of Kv6.3 subunits represents a compensatory mechanism directed to maintain a similar contribution of Kv currents to resting V_m_ (Moreno-Domínguez et al., 2009).

So far, VSMCs ion channels in the BPH model have been mainly studied in the mesenteric artery and that has been the focus of this review. Some of the changes described in this model, as the decreased activity of BK channels, have also been found in humans (Yang et al., 2013; Cheng et al., 2016) and in other models of hypertension (Tykocki et al., 2017). However, the comprehensive study of most of the expressed channels in this particular vessel has demonstrated the importance of having a global portrait of all the individual changes to interpret properly their complex interplay. Among the changes observed it is worth mentioning the different assembly of pore-forming subunits and the spatial organization of the ion channels, where we can find the paradox of higher local activity with a global downregulated expression due to the cooperative activity of clustered ion channels.

Obviously, the understanding of the role of ion channels in mesenteric VSMCs is just the tip of the iceberg to understand the BPH phenotype. Other vessels, other cells from the vessel wall and other organs and systems need to be studied. The phenotypic similarities to human essential hypertension and the existence of a normotensive (BPN) control strain make the BPH mice an excellent model to engage in the challenge of quantifying the little effects of the many changes associated with hypertension.
